# Research progress of *Auricularia heimuer* on cultivation physiology and molecular biology

**DOI:** 10.3389/fmicb.2022.1048249

**Published:** 2022-11-10

**Authors:** Xin Sun, Chunhui Yang, Yinpeng Ma, Jiechi Zhang, Lei Wang

**Affiliations:** ^1^Department of Biotechnology, Institute of Advanced Technology, Heilongjiang Academy of Sciences, Harbin, China; ^2^Institute of Microbiology, Heilongjiang Academy of Sciences, Harbin, China

**Keywords:** *Auricularia heimuer*, *Auricularia*, edible-medicinal fungus, large fungus, cultivation physiology, molecular biology

## Abstract

*Auricularia heimuer* (*A. heimuer* F. Wu, B. K. Cui, Y. C. Dai), a well-known gelatinous fungus used for both food and medicine, is a major edible fungus with a more than 1000-year history of cultivation in China. The nutrients of *A. heimuer* are abundant, including polysaccharides, melanin, mineral elements, etc. The *A. heimue*r polysaccharides exhibit antioxidant, immunomodulatory, and anticancer properties. *A. heimuer* is a completely different species grown in China, unlike *Auricularia auricula-judae* (Bull.) Quel, which was used to characterize it. The cultivated strain varies based on the local climatic factors and cultivation practices. Hardwood chips are the primary material utilized in the cultivation of substitute materials, which is the principal cultivation technique. However, in actual production, straw is frequently replaced for some wood chips to address the issue of a lack of wood. There are three different types of growing techniques: open-air ground cultivation, arch cultivation, and shed-type hanging substitute cultivation of these three, the quality of *A. heimuer* grown in a shed is superior to that grown in an open-air environment. The *A. heimue*r genome sequencing project started later than expected, and the entire genome sequencing was not finished until 2019. *A. heimuer*’s molecular biology studies have mostly concentrated on analyzing genetic diversity and identifying cultivars using molecular markers including RAPD, ISSR, and ITS. There have only been a small number of studies on the function of *A. heimuer* genes, which have only focused on the preliminary cloning and expression study of a few genes, including the laccase gene and the triterpene compound production gene, among others. However, there is still a lack of comprehensive information concerning *A. heimuer*, necessitating a synopsis. To our knowledge, this is the first published review of *A. heimuer*, and it summarizes the most recent studies on its molecular biology and cultivation. This review can serve as a guide for future research on the fungus.

## Introduction

**FIGURE 1:**
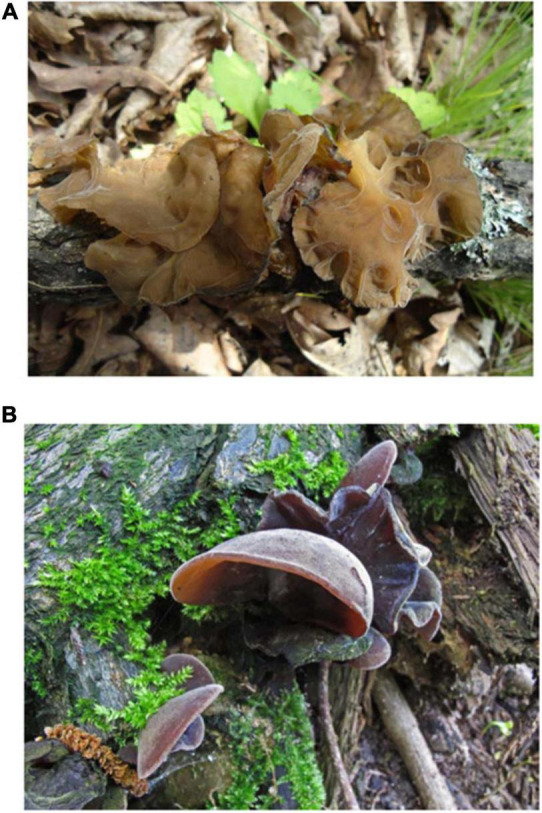
Basidiocarps of *Auricularia heimuer* and *Auricularia auricula-judae* (Wu and Dai, 2015). **(A)** Basidiocarps of *A. heimuer* (Dai 13788); **(B)** Basidiocarps of *A. auricula-judae* (LYBR 5404).

*Aricularia heimuer* (*A. heimuer* F. Wu, B. K. Cui, Y. C. Dai), a valuable large fungus used for both food and medicinal, is a member of the *Basidiomycota, Agaricomycetes, Auriculariales, Auriculariaceae*, and *Auricularia* (Kobayasi, 1981; Bai et al., 2021). It is an edible fungus that parasitizes on rotting and decaying wood. For more than a thousand years, it has been cultivated in China and utilized for food and medicine (Teng, 1939). *A. heimuer* was grown as a significant food source in Asia because of its high protein, trace element, vitamin, and carbohydrate content as well as its low fat content (Jonathan and Fasidi, 2001; Wu Z. C., 2017; Sun et al., 2021). It is used as medication for its anticancer, detoxifying, anticoagulant, hypoglycemic, and cholesterol-lowering properties (Fan et al., 2007; Luo et al., 2009). *A. heimuer* has a high carbohydrate content and the main component is the polysaccharide, which is one of the main active ingredients in it. It is a macromolecular active ingredient with 1,3-β-glucan as the main chain, and with mannose, the content can reach 64.19% (Huang et al., 2019). Polysaccharide has effects those can inhibit tumor growth as well as act as an anticoagulant and hypolipidemic agent (Song, 2011; Nguyen et al., 2012; Huang et al., 2019). An abundant natural pigment found in fungi names melanin has antioxidant, antibacterial, and immune-boosting properties. It is also a type of macromolecular compound with numerous potential uses (Li et al., 2020). The element Fe content of *A. heimuer* is 0.69 g/kg, about 49 times that of meat and 20 times that of spinach, the element Ca content is 5.2 g/kg, about 86 times that of meat. The protein content of *A. heimuer* is 10.62% and is plentiful in several amino acids, especially leucine and lysine. *A. heimuer* is a low-fat nutritious meal given that it only contains 0.2% fat (Gu et al., 2020). Additionally, water can be used to extract the flavonoids, which have an antioxidant function, and the *A. heimuer* also contains many vitamins such as carotene (Jonathan and Fasidi, 2001; Li G. et al., 2021). *Auricularia auricula-judae* (Bull.) Quel. was given to Chinese black fungus by Kalchbrenner and Thümen (1881), and it has since gained widespread acceptance. *Auricularia auricula-judae* (Bull.) Quel, however, was later discovered to be a species complex, according to further research (Wu et al., 2014, 2021; Wu and Dai, 2015; Fang W. et al., 2019). The common black fungus that was cultivated in China was a novel species that different from *A. auricula-judae.* It was given the name *Auricularia heimuer* F. Wu, B. K. Cui, and Y. C. Dai in 2014. *A. heimuer* differs in color, has smaller abhymenial hairs, basidia, and basidiospores than *A. auricula-judae* (Figure 1; Dai and Yang, 2008; Dai et al., 2010; Wu et al., 2014).

**FIGURE 2:**
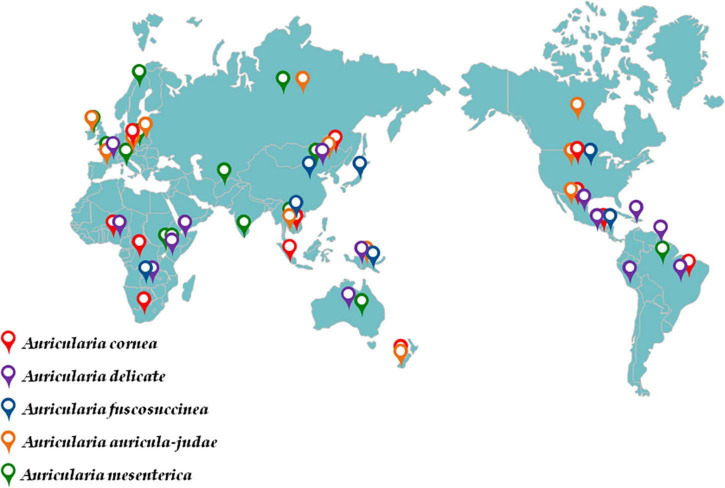
Global distribution of *Auricularia*.

Geographically, the wild *Auricularia* can be found in Asia, Europe, North America, and other temperate and subtropical regions (Figure 2; Table 1; Kobayasi, 1981; Wu et al., 2021). The techniques of harvesting have gradually changed from wild collecting to artificial cultivation as its nutritional and therapeutic potential has grown. In terms of production, China is the fourth-largest producer and exporter of black fungus, with northern China serving as the primary *A. heimuer* growing region. *A. heimuer* was produced in China in total in 2020 at a volume of 7,064,300 tons, with more than 40% of that production occurring in the north of China (Li W. F. et al., 2021; Xu et al., 2021). *A. heimuer* is mostly grown using two different techniques: wood cultivation and substitute cultivation. Substitute cultivation is more popular because it is less expensive than wood cultivation (Zhao et al., 2021). Although the wild resources in northern China are abundant and the climate is ideal for the growth of *A. heimuer*, the uncontrolled seed production and introduction into the production process have led to the chaos of its names and the degradation of several great qualities. The key to breeding is understanding its genetic relationship because, at the same time, its genetic history is extremely complex as a result of extensive artificial domestication and natural selection. The majority of fungus varieties are currently identified and distinguished using molecular markers like RAPD and SSR; however, researchers generally agreed that the stability of RAPD markers was insufficient and that many repeat tests were required to produce more reliable results (Li J. J. et al., 2021; Yin et al., 2022).

**TABLE 1:** Global distribution of *Auricularia*.

Complex name	Species name	Distribution regions (country)
*Auricularia*	*A. camposii*	Brazil
*cornea*	*A. cornea*	China, The Republic of Benin, Brazil, Germany, Ghana, South Africa, Singapore, Sri Lanka, Vietnam
	*A. eburnea*	China
	*A. eminii*	Democratic Republic of the Congo, South Africa
	*A. nigricans*	Costa Rica, Mexico, USA
	*A. novozealandica*	New Zealand
*Auricularia*	*A. australiana*	Australia
*delicata*	*A. conferta*	Australia
	*A. delicata*	Cameroon, Papua New Guinea
	*A. lateralis*	China
	*A. pilosa*	Australia, Ethiopia, Tanzania, Zambia
	*A. tremellosa*	Brazil, Peru, Mexico
	*A. sinodelicata*	China
	*A. scissa*	Dominican Republic
	*A. subglabra*	Brazil, Costa Rica, French, Guiana
*Auricularia*	*A. fibrillifera*	China, Papua New Guinea, Zambia
*fuscosuccinea*	*A. fuscosuccinea*	Brazil, USA
	*A. papyracea*	Japan
	*A. thailandica*	China, Thailand
	*A. xishaensis*	China
*Auricularia*	*A. americana*	Canada, USA, Mexico
*auricula-judae*	*A. angiospermarum*	USA
	*A. auricula-judae*	Czech Republic, France, Germany, UK
	*A. hainanensis*	China, Japan, New Guinea
	*A. heimuer*	China, Japan, Russia
	*A. minor*	China
	*A. minutissima*	China
	*A. tibetica*	China
	*A. villosula*	China, Russia, Thailand
*Auricularia*	*A. africana*	Kenya, Uganda.
*mesenterica*	*A. asiatica*	Thailand
	*A. brasiliana*	Brazil
	*A. mesenterica*	China, Czech Republic, Estonia, France, Italy, Russia, Switzerland, UK, Uzbekistan
	*A. orientalis*	China
	*A. pusio*	Australia
	*A. srilankensis*	Sri Lanka
	*A. submesenterica*	China

Genome sequencing information has significant ramifications for understanding the genetic basis, molecular mechanisms, and evolutionary mechanisms of species-specific biological features. The genetic research on edible and therapeutic fungi is considerably behind that of fungi of other species. Chen et al. (2012) completed the sequencing of *Ganoderma lucidum* in 2012, and *Lignosus rhinocerotis* (Cooke) Ryvarden had its whole genome sequenced in 2014 (Chen et al., 2012; Yap et al., 2014). In comparison to other edible fungi, the genome of *A. heimuer* has been studied relatively late, and the whole genome sequencing of *A. heimuer* was only completed in 2019 (Yuan et al., 2019). This review intends to give a thorough overview of the scientific data on the molecular biology and cultivation physiology of *A. heimuer* as a nutritious food and medicine and to serve as a guide for future research on the fungus.

## Progress of physiological research on cultivation of *Auricularia heimuer*

*Auricularia heimuer* is a unique edible mushroom artificial cultivation product in China and one of the edible mushroom species with independent intellectual property rights. It is also a unique edible mushroom species in Heilongjiang Province. Due to the distinctive resource characteristics, climatic conditions, and agricultural order conditions, Heilongjiang province’s *A. heimuer* production scale, production level, and product quality are at the forefront of China. This dominant trend will become increasingly clear as industrialization accelerates. In China, the artificial cultivation of *A. heimuer* primarily went through four stages: The initial stage used the natural inoculation method of spores, which was followed by two phases of spore liquid spraying and pure strain inoculation of linden wood. After the 1970s, with the progressive advancement of substitute material cultivation technology, it created the substitute material cultivation technology that is currently widely used (Zhang et al., 2009). The primary ingredient in the cultivation of *A. heimuer* substitutes is frequently hardwood chips. Hardwood contains 35–45% cellulose, 20–30% hemicellulose, and 15–20% lignin, which make up the majority of the plant cell wall. The latter two can build a spatial network structure by covalent bonding. The cellulose molecular chains aggregate into bundles and are arranged in an ordered manner to form a cell wall fibril framework, in which they are embedded. The three are closely connected and together form lignocellulose, which is the main source of carbon in the growth and development of *A. heimuer* (Shu, 2021).

A variety of nitrogen sources, mostly categorized as composite nitrogen sources, amino acid nitrogen sources, and inorganic nitrogen sources, can be used by edible mushrooms. According to research, organic nitrogen performs better than inorganic nitrogen in the practical application of growing edible mushrooms, compound nitrogen outperforms single component nitrogen, and ammonia nitrogen outperforms nitrate nitrogen. Additionally, the ratio of carbon to nitrogen has a big impact on how edible mushrooms grow and develop. The carbon/nitrogen ratio is best in the range of 20:1 for the growth stage of mycelium, where the nitrogen content demand is often higher, and in the range of 30–40:1 for the growth stage of fruiting bodies (Xiang, 1990). *A. heimuer* can utilize nitrate, urea, protein, ammonium, ammonia, and amino acids as nitrogen sources. Additionally, minerals like P, S, K, Ca, Mg, etc., are essential for the growth and development of edible mushrooms (Jonathan and Fasidi, 2001). At present, *A. heimuer* production in China mainly utilizes substitution cultivation technology, forming cultivation modes such as open-air ground-swing cultivation, small arch cultivation, and shed-type hanging bag cultivation. The three techniques of cultivation are managed in different ways, and the fungus quality varies slightly. In general, shed cultivation produces higher-quality fungus than open-air cultivation (Bian, 2006; Yao, 2017; Zhang et al., 2020).

Research on cultivation technology primarily examines applied research, such as formulation optimization, temperature and humidity optimization, and ventilation index, and focuses on cultivars, cultivation substrate formulation, disease control, etc. In 2020, by using antagonism tests and esterase isoenzyme analyses to verify the affinities of 24 strains, together with the results of mycelial growth and cultivation tests, Liu Y. Y. et al., 2020) chose the strains Black3 and M8, which were suitable for cultivation and promotion in Liaoning Province. In the same year, *A. heimuer* production trials were conducted by Chen et al. (2020) using five strains as test materials. After analyzing the physiological traits of each strain, it was shown that strain C10 could efficiently save money and was suitable for promotion and application (Chen et al., 2020). According to local meteorological circumstances, cultivation practices, and cultivation substrates, it was demonstrated that different production locations should choose high-quality and high-yielding strains fit for local cultivation.

In artificial *A. heimuer* production, the only source of nutrients is the cultivation substrate, which is essential for the quality and bioconversion rate of the organism. For the cultivation of *A. heimuer*, a variety of raw materials are suitable, such as hardwood chips, cottonseed hulls, bran, maize meal, soybean meal powder, corn cob, etc. (Li and Li, 2017; Shu, 2021). Hardwood chips and bran are frequently employed in the actual manufacturing of *A. heimuer* as the only sources of carbon and nitrogen in the traditional recipe (Zhang et al., 2020). However, as *A. heimuer* culture scales up, a severe hardwood chip shortage occurs; as a result, the role of strawization of cultivation substrate is particularly crucial for the growth of the sector (Table 2; Bian, 2006; Wang W. et al., 2015). Li and Li (2017) discovered that the cultivation substrate ratio of 32% corn straw, 18% corn cob, and 32% wood chips led to the outstanding growth of *A. heimuer* mycelium (Li and Li, 2017). Peng et al. (2018) demonstrated that cotton firewood could be used to cultivate *A. heimuer*, and the yield rose by 24.84% when the content of cotton firewood was 46.8% compared to the control group.

**TABLE 2:** Different formulations of *Auricularia heimuer* substitute cultivation.

Material type	Proportion (%)	Result	References
Lespedezain	60	Mycelium growth thicker, stronger, faster and the output was higher	Ma et al., 2014
Flax residues	30	Improve yield and biological efficiency of fruiting body	Wang J. H. et al., 2015
Corn straw	32	The average bag yield of fungus is 50.41 g, with higher economic efficiency	Li and Li, 2017
Cassava stalk sawdust	45	Significantly improve economic efficiency	Qin et al., 2017
cotton stalk	78	Fast and good growth of mycelium. High yield of fungus	Peng et al., 2018
Tea stalks and tea-branch dust	48.5	Higher yield and biological efficiency	Liu J. X. et al., 2020
Soybean straw	60	Higher level of crude protein, fiber and amino acid content	Pan et al., 2021
Mulberry, pear, peach and grape branches	10	Lower yield but higher amino acid content	Wang, 2011

The two basic components of edible mushroom illnesses are fruiting bodies and mycelial ailments. Red ears, distorted ears, rotten ears, etc., are common manifestations of *A. heimuer* fruiting body disorders. The pathophysiology of these lesions has not been well-studied (Liu et al., 2008). The primary illnesses of mycelial diseases are caused by invasive and competitive fungi (Sun and Bian, 2012). *Trichoderma* spp., *Penicillium* spp., *Aspergillus* spp., *Neurospora* spp., *Alternaria* spp., and *Mucor* spp. are the most frequent contaminating fungi in the production of *A. heimuer* replacements, and sterilizing is the key disease management method.

## Advances in molecular biology of *Auricularia heimuer*

### Application of molecular marker technology in the study of genetic diversity of *Auricularia heimuer*

Studies on the molecular biology of *A. heimuer* are very few, and the majority of the studies have concentrated on the use of molecular markers to study the genetic diversity of *A. heimuer* and so give a reference for variety identification. Initially, genetic diversity analysis and variety identification relied on RAPD and ISSR molecular marker approaches (Zhang et al., 2007; Tang et al., 2008). The use of rDNA-ITS and SRAP approaches for genetic diversity analysis in recent years has also increased. When combined with agronomic features, these techniques can offer a theoretical framework for the screening and selection of cultivated strains of *A. heimuer* (Yao et al., 2017).

Wu et al. (2004) isolated 52 monokaryotic F1 strains of the dikaryon H2J3 of *A. auricula* using single-spore isolation from the fruiting bodies. To ascertain whether the dikaryotic mycelia had formed, they conducted a mating experiment involving the F1 progeny and their parents, combined the method of dyeing the mycelia’s nuclei, and observed the nuclear phase under a fluorescence microscope as well as the clamp connection under a light microscope. All F1 progenies’ mating traits were also identified. According to the mating phenotype, the F1 progeny were split into two groups (F1-A1 and F1-A2), and the DNA of each strain in each group was blended in an equal ratio to create an isogenic pool of mating type genes. The amplification results were consistent across the F1 progenies and their parents, that are of the same mating type, according to RAPD analysis of 64 randomly chosen primers, which revealed that primer S126 amplified a particular band, S1261021, between the two pools. According to the findings, S1261021 is a molecular marker connected to the gene for the mating phenotype in *A. heimuer* (Wu et al., 2004).

*Auricularia heimuer* strains were distinguished using the RAPD and ISSR methods, and the test strains were divided into three groups based on their genetic kin (Xiao et al., 2006; Song et al., 2007). The results demonstrated the effectiveness of RAPD and ISSR approaches for the quick and precise identification of *A. heimuer* strains, and they were the best techniques for *A. heimuer* fingerprinting study. The applicability of the RAPD molecular marker technique for genetic diversity analysis of *A. heimuer* was also shown in a study by Li et al. (2007). They employed RAPD to analyze the genetic diversity of nine *A. heimuer* types in Heilongjiang province using 14 primers that were chosen from 40 primers and had good polymorphism. The results demonstrated the high genetic diversity of the nine types mentioned above. Liu et al. (2011) selected nine pairs of SRAP primers for the DNA of 18 wild strains and six cultured strains using the PCR-SRAP method. NTSYSpc software was used to examine the genetic diversity. The results of the cluster analysis revealed that the genetic similarity coefficient of *A. heimuer* could be divided into five groups and was at a level of 0.63. Additionally, the SRAP marker technology revealed significant genetic differences between cultivated and wild strains, proving the viability of using SRAP markers for the analysis of genetic diversity (Liu et al., 2011).

The genetic diversity of 17 commercially available *A. heimuer* cultivars was examined in Heilongjiang Province’s eastern region in 2019 using the ITS sequence analysis method (Li R. R. et al., 2019). The results indicated the genetic diversity of commercially accessible *A. heimuer* in the eastern section of Heilongjiang Province was substantial and that the 17 *A. heimuer* species investigated could be divided into seven categories. However, it was also discovered that some varieties with different names had evolutionary distances of their ITS sequences that were the same. This suggests that there have been instances of commercially available *A. heimuer* strains with different trade names that are actually the same variety and that the names of market varieties are unclear. Liu Y. Y. et al. (2020) used 15 cultivars as test materials to examine the genetic diversity of 15 cultivars of *A. heimuer* using the strain affinity test, rDNA-ITS, and SRAP clustering analysis. The results indicated that *A. heimuer* could be successfully clustered using both ITS and SRAP approaches, which produced comparable outcomes (Liu Y. Y. et al., 2020).

For a more accurate characterization of the genetic diversity of *A. heimuer*, rDNA-ITS and SRAP along with strain-specific affinity tests could be used. *A. heimuer* cultivars had minimal polymorphism, and some of the genetic changes between several kinds were minor, according to the identification results. By combining mycelial growth rate, yield, and agronomic characteristics of the substrates, Song et al. (2021) used SSR technology in 2021 to analyze the relatives of 15 *A. heimuer* strains and to screen out high-quality and high-yielding strains suitable for cultivation in southern regions of China. Additionally, the relationship between their agronomic characteristics and yield was examined (Song et al., 2021).

### Functional study of genes related to *Auricularia heimuer*

*Auricularia heimuer* genes were the subject of few investigations, and this research merely cloned and examined the expression of a few genes without further investigation of the genes’ activities. By employing degenerate primers br1 -F and br1 -R, which were designed based on the conserved amino acid sequence of a STE3 pheromone receptor in *Schizophyllum* commune, Xiao et al. (2006) obtained an 811 bp length pheromone receptor gene fragment in 2006.

In 2014, seven laccase genes were cloned from the *A. auricula-judae* strain Au916 and used by Fan et al. (2014) to study the expression of laccase genes during the production of fruiting bodies. A phylogenetic analysis was also carried out. They discovered that the function of the laccase genes from *A. auricula-judae* differed noticeably from that of other basidiomycetes, and that the expression patterns of seven laccase genes varied as well (Fan et al., 2014). Based on the transcriptome and genome of *A. auricula*, Zou et al. (2020) employed bioinformatics in 2020 to examine the important enzyme genes in the synthesis of triterpenoids. The findings demonstrated the identification of 14 potential genes, including four *AACT* genes, one *HMGS* gene, one *HMGR* gene, one *PMK* gene, three *FPPS* genes, one *SQS* gene, and three *LS* genes, that are involved in the production of triterpenoid MVA. Two metabolic pathways involved in the production of triterpenoid were annotated in 93 unigenes, or roughly 0.82% of the total. They looked further into the potential candidate genes for triterpenoid production in *A. auricula* by examining the homology of the selected genes (Zou et al., 2020).

Zhang (2020) selected 12 candidate internal reference genes and designed primers across introns using samples of different strains of *A. heimuer* (A14, A137, and A12) and different fertility stages (mycelium, primordium, and fruiting stages) as experimental materials. The qRT-PCR technique was used to amplify the genes, and geNorm, NormFinder, BestKeeper, and ΔCt algorithms as well as the comprehensive evaluation software RefFinder were used to screen the suitable internal reference genes. The results showed that 18S rRNA, β-TUB, EF1-a, and 28S rRNA were suitable as internal reference genes for different strains, and APRTase, 18S rRNA, and 28S rRNA were suitable as internal reference genes for different fertility stages. qRT-PCR was used to analyze the differential expression of key enzyme genes (*PGI*, *PGM*, and *UGPase*) for polysaccharide synthesis in *A. heimuer* under different experimental conditions. The results showed that the relative expression of *UGPase* was higher in the fruiting stage than in the primordium and mycelium stage, and the relative expression of *PGI* and *PGM* was higher in the mycelium stage than in the primordium and fruiting stage. The relative expression of *UGPase* in glucose and peptone was 1-fold and 5-fold higher than that of the control, and the relative expression of *PGM* in different nutrient conditions was lower than control. The relative expression of *PGI* in different stress conditions such as 4°C, 30°C, and pH = 9 was 10-fold, 4-fold, and 6-fold higher than that of the control. The relative expressions of *UGPase* and *PGM* were lower than that of the control in different stress conditions.

### Genomic study of *Auricularia heimuer*

With the rapid development of gene sequencing technology and the decreasing cost of sequencing, more and more fungal genomes have been sequenced, and the study of fungal genomes is important to reveal the genetic basis, molecular mechanism, and evolutionary mechanism of fungal biological traits. Goffeau et al. (1996) completed the whole genome sequencing of *Saccharomyces cerevisiae*, which was the first eukaryotic organism to complete the whole genome sequencing, and subsequently, many other fungal genomes were also sequenced. However, whole-genome studies of large edible fungi of the *Stenotrophomonas* phylum have lagged far behind other types of fungi.

Floudas et al. (2012) completed the whole genome sequencing and assembly of *Auricularia subglabra*, which has a genome size of 74.92 M and a GC content of 57.9%, encoding 23,783 genes, 23,555 proteins, and 226 tRNAs. In April 2017, Jilin University completed the whole genome sequencing of *Auricularia auricula-judae*, which had a genome size of 43.57 M and a GC content of 56.6% (Yuan et al., 2019). Li et al. (2018) completed the whole genome sequencing of *Auricularia polytricha*, which had a genome size of 38.69 M and a GC content of 46.7%. In 2019, the researchers from Jilin university of China completed the whole genome sequencing of *Auricularia cornea* with a genome size of 78.5 M and GC content of 59.5%. In the same year, Yuan et al. (2019) from Beijing Forestry University completed the whole genome sequencing, and obtained an *A. heimuer* genome of 49.76 M with a GC content of 56.98%, the number of coding genes was 16,244, and a total of 15,135 genes were functionally annotated, accounting for 93.17% of the total genes, which was also the first genome assembly and annotation analysis of *A. heimuer*. Fang M. et al. (2019) finished the mitochondrial sequencing of *A. heimuer* in 2019. The total size of its mitochondrial genome is 40,586 bp. It has 48 genes, 25 of which code for proteins, 22 for tRNA, and 1 for RNase P RNA (Table 3). The percentage of GC is 37.92% (Fang M. et al., 2019).

**TABLE 3:** Genomes of different species of *Auricularia*.

Species	Date	Total length (M)	GC%	Assembly level	References
*Auricularia subglabra*	2012.06	74.9202	57.9	Scaffold	Floudas et al., 2012
*Auricularia auricula-judae*	2017.04	43.57	56.6	Scaffold	Yuan et al., 2019
*Auricularia heimuer*	2017.12	49.76	56.98	Scaffold	Yuan et al., 2019
*Auricularia polytricha*	2018.07	38.6887	46.7	Scaffold	Li et al., 2018
*Auricularia cornea*	2019.09	78.5041	59.5	Contig	Yuan et al., 2019

## Conclusion and future prospects

*Auricularia heimuer* offers a wealth of nutritional and therapeutic benefits and is high in polysaccharides, amino acids, vitamins, calcium, iron, phosphorus, and other minerals. The polysaccharides of *A. heimuer* have anticancer, antioxidative, hypolipidemic, and immunomodulatory properties (Fan et al., 2007; Luo et al., 2009; Nguyen et al., 2012). Its fruiting bodies’ gum also has a significant capacity for adsorption and, as a result, has a lubricating effect on the intestines. People are paying more and more attention to a healthy diet, selecting healthier and more nutritious ingredients, and becoming more aware of health care as their material standard of living has improved. Due to its high nutritional value and low fat content, *A. heimuer* is a nutritious meal with enormous potential, and as a result, its sales volume has greatly expanded (Lv et al., 2022; Yang et al., 2022). Research on *A. heimuer* is not very well-developed elsewhere. According to the species, edible mushroom research is primarily concentrated in other countries on edible mushroom species like *Agaricus bisporus* and *Lentinula edodes* that have more established industries. Based on the industry, it appears that their edible mushroom production is primarily factory-based, with an emphasis on seed breeding, specialized production technologies, etc. There are less studies on *A. heimuer*, and those that do tend to concentrate on the methods for extracting and purifying polysaccharides and other powerful substances, as well as the investigation of immunological activity and other effects. In addition to having a large-scale production of *A. heimuer*, China also possesses outstanding quality. The primary method of *A. heimuer* cultivation at the moment is substitute cultivation, but as the scale of the cultivation increases, the conflict between the mushroom and the forest becomes more and more serious (Zhang et al., 2015; Chen et al., 2020). As a result, most substrate formulations now use corn straw, soybean straw, and corn cob in place of wood chips, and the scarcity of hardwood chips can be addressed by scientific weighing while maintaining quality.

(1) Substrates for cultivation. In recent years, a number of researchers have grown edible mushrooms on cultivation substrates like herbs, grasses, tea stalks, and fruits (Liu J. X. et al., 2020). This has increased the variety of materials available for cultivation, lowered production costs, and aided in the full utilization of agricultural resources (Wang, 2011). Wu S. F. (2017) used mycorrhiza and bagasse instead of certain wood chips to culture fungus. The results revealed that when the ratio was 21% for mycorrhiza and 21% for bagasse, it might promote mycelial growth (Wu S. F., 2017). Qin et al. (2017) discovered that cassava straw could considerably improve the mycelial growth rate of *A. polytricha* with no discernible change in yield from the control group when utilized as the primary material to culture *A. heimuer*. (2) Improving the quality and yield of *A. heimuer*. The quality and growth of *A. heimuer* can be effected by the type of cultivation substrate (Pan et al., 2021), in addition, in 2020, a study from Zheng et al. (2020) showed that *A. heimuer* cultivated in bags with sawdust medium under moso bamboo forest boasts uniform size/good glossiness and less impurities. Compared with the control group, the yield and nutritional quality of *A. heimuer* cultivated under bamboo forest were both high, indicating that in addition to the substitute substrate, the environment and different cultivation methods have a great influence on the quality and yield of *A. heimuer*. Cultivation of *A. heimuer* under bamboo forest can effectively improve the space utilization of forestland and maximize the benefits. The space usage of forestland can be successfully improved through the cultivation of *A. heimue*r under bamboo forests, maximizing the advantages (Zheng et al., 2020). (3) Processed products of *A. heimuer*. The majority of *A. heimuer*’s processed products, such as the powder and freeze-dried flakes as well as beverages containing other fruits, remain in the rough processing stage. *A. heimuer*’s deep processing still needs to be improved, and the level of processing needs to be raised to create products more suited for the modern market while preserving as much of the nutritional and therapeutic benefits of the its original active components.

The lengthy domestication process of *A. heimuer* has resulted in categorization confusion among its several cultivars, making it challenging to tell them apart (Tang et al., 2008; Yuan, 2018). The sequencing of *A. heimuer*’s genome is crucial for identifying different cultivars of the bacterium, but the work on this genome only recently began, and there are only a few fundamental studies on the molecular biology of *A. heimuer* (Bai et al., 1998; Bian et al., 2000). The majority of these studies concentrated on the initial work on the molecular identification of *A. heimuer* strains using molecular markers, the cloning and expression analysis of some genes, and there are few research focused on the mechanism. The number of coding genes and genome size of the various *Auricularia* species varies, and they are not very able to refer to one another (Dai et al., 2019; Fang et al., 2020). Li X. et al. (2019) conducted a transcriptome analysis of the *Auricularia cornea* in selenium accumulation. As a result of the incomplete whole genome sequencing, there are still gaps in the studies on the transcriptome analysis of *A. heimuer* (Li X. et al., 2019). Even though the full genome of the genus *Auricularia* has only been partially explored, all the genomes were sequenced and assembled at the scaffold or contig level rather than the chromosome level, which also restricts some later evolutionary analyses like chromosome localization analysis and covariance analysis. As a result, the deep genome sequencing of *A. heimuer* will aid in the study of functional genomics and serve as a starting point for its molecular breeding studies.

## Author contributions

XS: conceptualization, formal analysis, investigation, data collection, and writing—original draft. LW and JZ: supervision. CY and YM: data collection. All authors contributed to the article and approved the submitted version.
